# Validation of a Modified Algometer to Measure Mechanical Nociceptive Thresholds in Awake Dogs

**DOI:** 10.1155/2015/375421

**Published:** 2015-05-05

**Authors:** Ubedullah Kaka, Hui Cheng Chen, Yong Meng Goh, Adamu Abdul Abubakar, Sharida Fakurazi, Mahdi Ebrahimi

**Affiliations:** ^1^Department of Veterinary Clinical Studies, Faculty of Veterinary Medicine, Universiti Putra Malaysia, 43400 Serdang, Selangor, Malaysia; ^2^Department of Surgery and Obstetrics, Faculty of Animal Husbandry & Veterinary Sciences, Sindh Agriculture University Tandojam, Sindh 70060, Pakistan; ^3^Department of Veterinary Preclinical Sciences, Faculty of Veterinary Medicine, Universiti Putra Malaysia, 43400 Serdang, Selangor, Malaysia; ^4^Laboratory of Vaccines and Immunotherapeutics, Institute of Bioscience, Universiti Putra Malaysia, 43400 Serdang, Selangor, Malaysia; ^5^Department of Human Anatomy, Faculty of Medicine and Health Science, Universiti Putra Malaysia, 43400 Serdang, Selangor, Malaysia

## Abstract

This study was conducted to validate the use of a modified algometer device to measure mechanical nociceptive thresholds in six dogs. Dogs were administered morphine intravenously (IV) at 1 mg/kg or saline at equivolume in a crossover design with one-week washout period. Mechanical nociceptive thresholds were determined before, after the administration of treatments at 5 minutes, and hourly for 8 hours. Thresholds were recorded at the carpal pad, metacarpal foot pad, tibia, femur, and abdomen. Heart rates, body temperature, and respiration were recorded at similar time points. Thresholds increased significantly (*P* < 0.05) from baseline values for up to 3 hours at tibia and abdomen, 4 hours at metacarpal pad, and 5 hours at the carpal pad and femur. Hypothermia, bradycardia, and change in respiration were observed in all dogs after morphine injection. Saline did not alter any threshold levels during the eight-hour study period, indicating no evidence of tolerance, learned avoidance, or local hyperaesthesia. The device and methods of testing were well tolerated by all the dogs. Results suggest that the modified algometer and method of application are useful to measure nociceptive mechanical thresholds in awake dogs.

## 1. Introduction

Painful stimuli in humans have been shown to induce similar physiological and behavioural changes in other nonhuman mammals [1]. In humans, the ability to communicate verbally and physically helps clinicians and researchers to assess pain. In animal patients, the task of pain assessment may be more difficult and challenging. Yet, a reliable method to assess and quantify pain is a prerequisite to assess the efficacy of analgesics and improve pain management.

Objective evaluation of pain in animals is a challenge to both researchers and clinicians [2]. A valid objective method of assessing pain would be of great advantage for the evaluation of analgesics and pain management in animal patients [3]. In dogs, the electrical [4, 5], thermal [6], and mechanical [7–9] noxious stimulation have been evaluated as objective methods to assess pain. The mechanical stimulation is the most practical method for the quantification of pain or subsequent hyperalgesia; this method is called algometry [7, 10, 11]. Algometry has been used to quantify abnormal pain thresholds and to assess effect of analgesics in orthopaedic [12] and soft tissue [13] conditions in humans. These algometry methods can stimulate both the A*δ* and C fibers, which are responsible for encoding clinical pain [14].

The algometers used in various studies involve applying pressure with a probe over the testing points on the animal body [3, 7–9]. These devices consist of a probe with tip or disc, which is applied perpendicular to the skin in gradual increasing force to produce noxious stimulus. Force applied to the tip of the probe is transmitted to a load cell and a voltage output is produced. The signal is transduced and amplified and the output is displayed in newtons or kilograms.

Few studies have reported the use of algometry in dogs [7–9, 15]. These studies used algometers to assess the postoperative efficacy of the analgesics following surgical procedures. KuKanich et al. (2005) described the use of an electronic von Frey device to study the pharmacodynamics of morphine [16]. Both the electronic von Frey and algometer work on similar principle. The advantage of an algometer over the electronic von Frey device is that it is a single and simple handheld unit without any electric wire or other parallel unit. It can be applied easily in the clinical setting in awake dogs compared to the electronic von Frey device.

This paper reports the use of a modified algometer and validation of the mechanical threshold determination technique in awake dogs. The hypotheses were that (1) mechanical nociceptive thresholds would increase after administration of morphine and (2) mechanical nociceptive thresholds would not change over time in control group.

## 2. Material and Methods

### 2.1. Animals

This study was subjected to the review and approved by the Universiti Putra Malaysia Animal Care and Use Committee (UPM/IACUC/AUP-R023/2013). Six healthy adult female dogs weighing 16.66 ± 2.65 (mean ± SD) were used in this study. Dogs were judged healthy based on physical examination, hematology, and blood biochemistry. Study was conducted in a randomized crossover design with one-week washout period between the treatments.

### 2.2. The Algometer Device and Its Modification

Wagner algometer (FPX 25, Wagner Instruments, Greenwich CT, USA) was used in this study. It consists of a soft-grip handle and piston with a pressure-sensitive strain gauge transducer with flat circular rubber tip probe of 1 cm diameter. The probe is incorporated with a load cell. The force applied to the end of the probe is transmitted to the load cell and a voltage output is produced. This voltage output is taken to a transducer and amplifier and is shown on the numerical LCD display in newtons (N). The pressure in lbf, kgf, N, or ozf is then recorded as mechanical thresholds at the time of reaction by animal.

**Figure 1:**
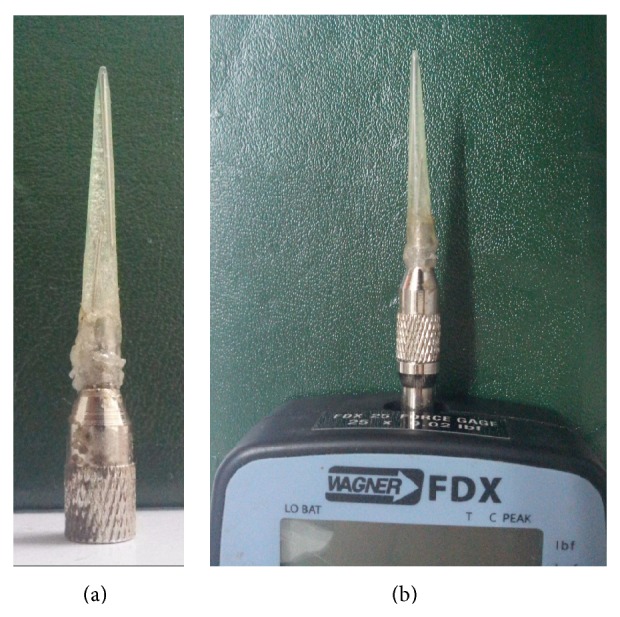
Tip made from 200 *μ*L polypropylene micropipette tip filled with epoxy and mounted on a modified hook (a). The tip-mounted hook was screwed onto the algometer (b).

The supplied 1 cm diameter rubber tip was evaluated in four dogs at abdomen, femur, and carpal pad. This tip required pressures exceeding half the capacity of the algometer, and most of the time, did not induce any withdrawal response. Therefore, we constructed a 1 mm diameter tip for the present study. The tip was made from 200 *μ*L polypropylene micropipette tip (Figure 1). The tip was strengthened by placing shafts of one 23 G and two 26 G hypodermic needles in the center, and the remaining space was filled with epoxy (5 Minutes Clear Epoxy Compound, Hardex Corporation). The algometer hook was straightened and shortened to facilitate mounting of the micropipette tip. Immediately after filling the tip with epoxy, it was mounted onto modified hook and left for hardening. The mounted hook was then screwed onto the algometer [17]. This custom-made tip stimulates an area of 0.7855 mm^2^ and exerts a pressure of 1273 kPa at 1 N force. The supplied tip stimulates an area of 78.55 mm^2^ and exerts a pressure of 12.73 kPa at 1 N force. These values for area and pressure were derived according to the following formula of area (*A* = *πd* ^2^/4) and pressure (*P* = *F*/*A*) in kPa.

### 2.3. Threshold Testing

**Figure 2:**
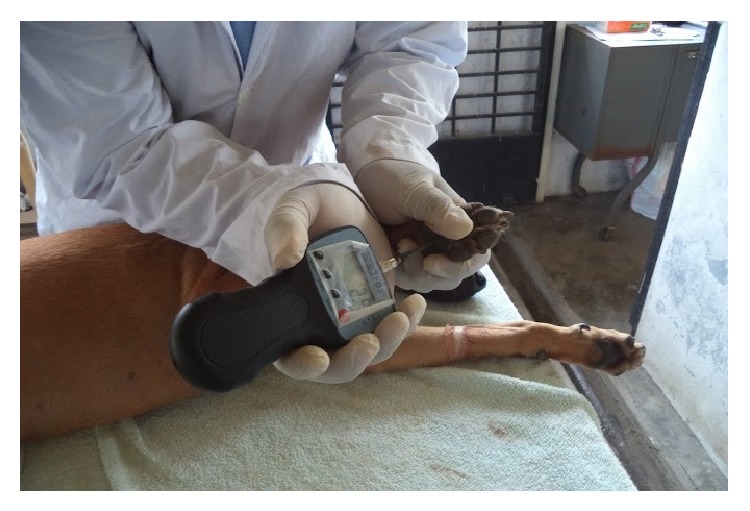
Mechanical nociceptive threshold measurement at metacarpal footpad of the right limb.

Researcher practised constant rate of force application of the algometer before the experiment. Dogs were habituated to the researchers and testing procedures for four weeks before the actual experiment. Researchers learned to identify the threshold responses of individual dogs during these trials. Measurements during the experiment were made with dogs lying on left recumbency with minimal or no restraint (Figure 2). According to Le Bars et al. (2001), in order to avoid tissue damage, cut-off pressure should be set at three times the thresholds of control [14]. Preliminary trials revealed that the thresholds at various body points were 5 to 6 N. Thus, the cut-off pressure was set at 18 N for this experiment.

A preliminary evaluation performed on the carpal pad, metacarpal footpad, tibia and femur of both right and left limbs, and abdomen in 5 dogs showed no difference in thresholds obtained from the right or left limbs [17]. Repeated measures did not decrease thresholds when performed on the next consecutive weeks. Therefore, in this experiment, thresholds were determined only on the right limbs with dogs lying on left recumbency and with one-week interval between treatments.

The carpal pad and metacarpal pad were tested by applying the tip of the algometer at midpoint of these pads (Figure 2). The tibia was tested on the distal laterodorsal surface where bone could be palpated through the skin. The femur was tested on the distal laterodorsal surface where bone could be palpated through the skin by displacing the muscle with little pressure applied with fingers. The abdomen was tested on the midline at the midpoint between umbilicus and pubis. The order of testing was metacarpal pad first, followed by carpal pad, femur, tibia, and abdomen. At each testing point the tip was placed at 90° angle to the surface area (Figure 2). Reaction upon first touch of the tip to the body points was considered a simple withdrawal reflex and not the threshold. Consistent increasing pressure was exerted until a withdrawal or escape movement was made, or the dog vocalized, whichever earlier.

Morphine (Hameln Pharmaceuticals, Germany) at 1 mg/kg or saline at equivolume was administered IV over 30 seconds. Thresholds were taken before administration of drug as baseline, after drug administration at 5 minutes, and hourly for 8 hours. The same operator applied the tip of the algometer at consistent increasing pressures until the dogs reacted. At each time point, thresholds at each location were determined in replicate, with one-minute interval between each measurement. Visual examination for tissue damage caused by pressure from algometer tip was performed during the testing periods and 24 hours after the completion of test. Each of the five body points was examined for redness, swelling, bleeding, exudates, and pain upon palpation. Dogs were assessed for any signs of lameness.

### 2.4. Statistical Analysis

The results are presented as means ± standard error of the mean (SE). Statistical analysis was performed using the SAS software package, version 9.3 (SAS Institute Inc., Cary, NC, USA). Prior to the analysis, data was checked for their conformance to the normal distribution using Shapiro-Wilk normality test. Changes in pain measurements over time and across treatment groups were compared using the repeated-measures ANOVA model. Values of *P* < 0.05 were considered significant; Bonferroni-adjusted *P* values were used when indicated by a significant *F* test (*P* < 0.05).

## 3. Results

**Figure 3:**
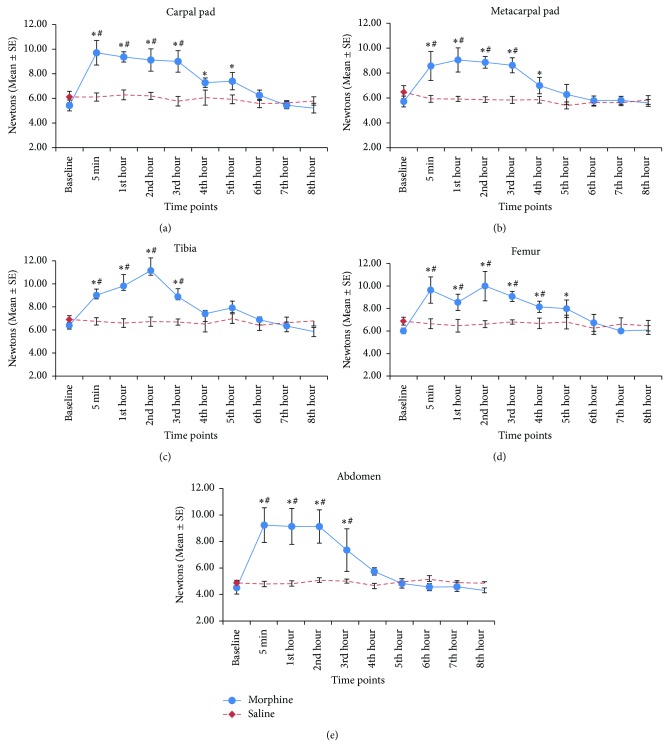
Mechanical nociceptive thresholds measured at five body points following saline or morphine treatment in a crossover design using 6 dogs (*n* = 5 at abdomen). Data are expressed as mean ± SE. ∗ denotes significant difference from baseline, # denotes significant treatment difference at corresponding time points, and *P* < 0.05.

The baseline threshold values (mean ± SE) at the carpal pad, metacarpal footpad, tibia, femur, and abdomen before administration of saline were 6.1 ± 0.45, 6.5 ± 0.51, 6.9 ± 0.31, 6.9 ± 0.35, and 4.9 ± 0.21 N, respectively, and 5.4 ± 0.44, 5.7 ± 0.43, 6.3 ± 0.31, 6.0 ± 0.25, and 4.52 ± 0.49 before administration of morphine. There was no treatment difference in the baseline threshold values. Algometer mechanical thresholds increased significantly after administration of morphine. Thresholds increased significantly from baseline values for up to 5 hours at the carpal pad and femur, 4 hours at metacarpal pad, and 3 hours at tibia and abdomen. On the contrary, saline did not produce significant change in the thresholds during the eight-hour study period at all the five body points (Figure 3).

**Figure 4:**
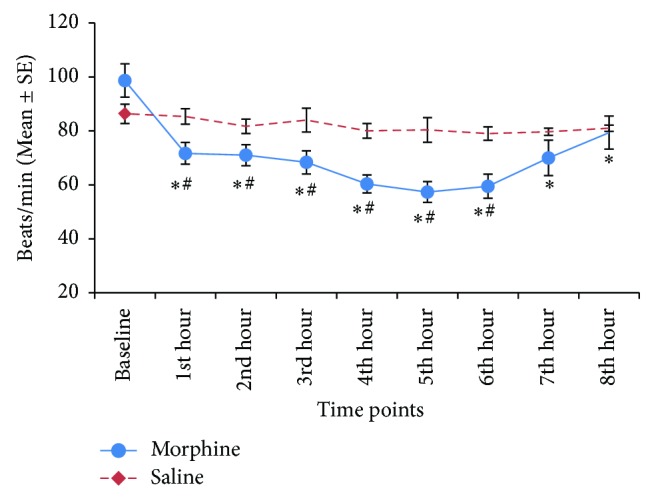
Heart rates of 6 dogs after administration of morphine (1 mg/kg IV) and saline at equivolume. Data are expressed as mean ± SE. ∗ denotes significant difference from baseline, # denotes significant treatment difference at corresponding time points, and *P* < 0.05.

**Figure 5:**
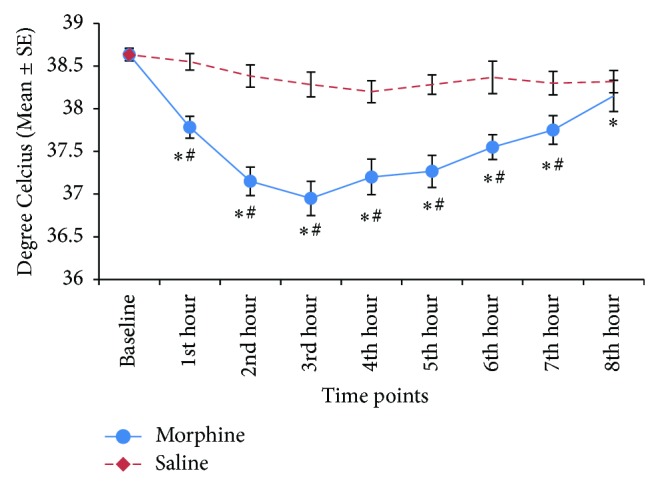
Body temperature of 6 dogs after administration of morphine (1 mg/kg IV) and saline at equivolume. Data are expressed as mean ± SE. ∗ denotes significant difference from baseline, # denotes significant treatment difference at corresponding timepoints, and *P* < 0.05.

Effects of morphine and saline on heart rates are depicted in Figure 4. Heart rates decreased significantly (*P* < 0.05) from the baseline values after administration of morphine. The maximum decrement in heart rates was recorded at the 5th hour after the morphine administration. Similarly, body temperature decreased significantly (*P* < 0.05) after morphine administration, with maximum decrement recorded at the 3rd hour (Figure 5). Five dogs developed rapid shallow breathing while one dog only exhibited mild increase in respiratory rate following morphine administration. Respiratory rates gradually reduced to acceptable range by 4 hours in 3 dogs, while the other 3 continued to pant throughout the eight-hour study period. Saline did not alter the heart rates, body temperatures, nor breathing patterns.

One of the dogs did not respond to application of the algometer up to the cut-off point of 18 N at its abdomen during the preliminary tests and, thus, was not tested in both morphine and saline for abdomen. The algometer device was well tolerated by all animals. Transient dimples were observed at the carpal pad, metacarpal footpad, and abdomen, which disappeared within 1 minute, before the replicate measurement. There was no sign of redness, swelling, bleeding, exudates, and lameness during, immediately after, and 24 hours after the experiment.

Following IV injections of treatments, dogs were placed on lateral recumbency to determine the interval to spontaneous righting. With saline injections, all six dogs immediately right themselves into sternal recumbency the moment researchers went away from them. Following morphine injections, one dog returned to sternal recumbency after 8 minutes, while the other 5 dogs returned after 20 minutes. All dogs developed miosis and mild sedation after morphine. None of the dogs vomited, but three dogs hypersalivated, one defecated, and four howled intensely for around 15 seconds after morphine injections.

## 4. Discussion

In this study, we have demonstrated successful use of a modified algometer to measure mechanical thresholds at five body points in dogs. Thresholds increased significantly following administration of morphine, while saline did not alter the thresholds. Baseline threshold values before morphine or saline treatment were comparable and not different, suggesting that the method of threshold measurement in this study is repeatable. After administration of saline, thresholds did not reduce, implying that the dogs did not develop local hyperaesthesia, learned avoidance or tolerance over the eight-hour study period.

In this study, mechanical thresholds increased significantly from baseline values for up to 3 hours at the tibia and abdomen, 4 hours at the metacarpal pad, and 5 hours at the carpal pad and femur. Results from the present study were comparable to previous study on mechanical and thermal thresholds in dogs. Kukanich et al. (2005) showed that morphine at 1 mg/kg IV increased electronic von Frey thresholds for up to 4 hours at the carpal pads [16]. Wegner et al. (2008) reported significant increase in the hind paw latencies to thermal stimuli for up to 4 hours after 1 mg/kg morphine, IV [18]. These data suggest that morphine at 1 mg/kg, IV, should increase both mechanical and thermal thresholds of the limbs for the duration of 4-5 hours. The difference in the duration of elevated thresholds at different body points in the present study is likely related to their difference in distribution and type of nociceptors, tissue thickness, and innervations. It is likely that the skin over the abdomen has more innervations compared to the carpal pad.

The ambient temperature has been reported to affect mechanical thresholds. In sheep, mechanical thresholds were found to be higher at ambient air temperature below 8°C [19]. The authors suggested that increase in mechanical thresholds was due to vasoconstriction and ischemia of nerve fibers induced by cold. The effects of temperatures on mechanical thresholds in dogs have not been reported. In the present study, the ambient temperature varied between 26°C and 32°C over the eight-hour experimental period. However, consistency of thresholds throughout the experiment in the saline treatment suggests that the mechanical thresholds were not affected by changes in the ambient temperature within this range.

Changes in the environment could result in higher variability in the thresholds measured [20]. In the present study, animals were trained before the actual experiment. These animals were well familiarized to the researchers, the environment of the testing station, and the testing procedures. Thus, variation in the environmental factors was minimized. The consistency of the thresholds in the saline treatment showed that this factor was well controlled in the study.

It may not be easy to fix an end-point of a nociceptive test, as there may be species and individual variation. Skin thickness, blood flow, and distribution of the nociceptors may affect the peripheral perception of stimulus [21]. It is therefore necessary to evaluate nociceptive threshold and identify the end-point for each species and individual to get optimal results [2]. For this purpose, we trained the animals before the experiment and identified end-points expressed by individual animal. The most common end-points were clear withdrawal reflex of the limb, vocalization, withdrawal of the limb accompanied with vocalization and guarding of the abdomen. In one dog, the algometer reading reached the cut-off point of 18 N, and it did not show any response at its abdomen. Therefore, abdomen testing was not included for this dog during the experiment.

The rate of force application could influence threshold measurements, where faster rates would result in higher readings [22]. In this study, the operator that applied the algometer had practised consistent application of force before the experiment. During thresholds determination, the operator did not look at the reading of the algometer as he applied consistent force. Instead, he concentrated on the dogs' response and immediately stopped when there was a withdrawal or escape response, or vocalization. The algometer has a “hold” facility that could record the last force applied. The end-points were cross-checked by another observer who took note of both the algometer readings and dogs' response. It would be desirable to have a meter that could continuously show the rate of force application to better guide the operator. Despite this deficiency, the consistent thresholds in the saline treatment suggest that the operator was consistent in applying the force.

A limitation of this study is its open label. The observers were not blinded to the treatments since recognisable effects of morphine compared to saline were expected. As described in the results, the euphoric and sedative effect, prolonged lateral recumbency time, and altered physiological parameters easily distinguished morphine from saline injections. Nevertheless, we attempted to minimize the potential bias by blinding the operator of the algometer to the threshold values, while another observer recorded the values displayed on the LCD screen.

Rise in the nociceptive thresholds after administration of an analgesic agent suggests antinociceptive effect of the agent [23]. In this study, we have demonstrated that the modified algometer and technique of application could clearly differentiate morphine from saline treatment. Therefore, this technique can be used to determine if a compound has antinociceptive effect. It also fulfils the characteristics described by Beecher (1957) [24], where the technique should be repeatable, reliable, and easy to apply with a clear end-point. The simplicity of this algometer also makes it practical to detect postsurgical hyperalgesia and monitor treatment response in the clinical setting.

## 5. Conclusion

In conclusion, the modified algometer and method of application described in this study can be used to determine mechanical nociceptive thresholds in awake dogs. Obvious elevation of the thresholds following morphine, compared to the consistent thresholds in saline treatment, validated the described technique for the study of antinociception in awake dogs.
